# Automated hemodynamic resuscitation system for computer-controlled norepinephrine, vasopressin, and fluid therapy in endotoxin-induced shock: a feasibility and physiological proof-of-concept study

**DOI:** 10.1186/s40635-026-00977-3

**Published:** 2026-09-20

**Authors:** Kazumasu Sasaki, Kazunori Uemura, Hiroki Matsushita, Yuta Nakamura, Kenta Ohba, Yasuyuki Kataoka, Takuya Nishikawa, Kei Sato, Hidetaka Morita, Masahiro Otake, Nana Hiraki, Masafumi Fukumitsu, Toru Kawada, Kenji Sunagawa, Joe Alexander, Keita Saku

**Affiliations:** 1 Department of Anesthesiology, Akita Cerebrospinal and Cardiovascular Center, Akita, Japan; 2 NTTR-NCVC Bio Digital Twin Center, National Cerebral and Cardiovascular Center Research Institute, Suita, Japan; 3 Department of Cardiovascular Dynamics, National Cerebral and Cardiovascular Center Research Institute, Suita, Japan; 4 Department of Anesthesiology, Shonan Kamakura General Hospital, Kamakura, Japan; 5 Medical and Health Informatics Laboratories, NTT Research, Inc., Sunnyvale, United States of America; 6 Circulatory System Research Foundation, Tokyo, Japan

**Keywords:** Vasoplegic shock, Endotoxin shock, Automated hemodynamic resuscitation, Vasopressin, Minimally invasive cardiac output monitor, Machine-learning analysis, Circulatory equilibrium framework, Physiology-driven resuscitation

## Abstract

**Background:**

To support hemodynamic management in vasoplegic shock, we previously developed a closed-loop automated infusion system of norepinephrine (NE) and fluid to automatically restore arterial pressure (AP) and cardiac output (CO). To enhance the practical feasibility and efficacy of the system, we integrated a minimally invasive CO monitor and a closed-loop control of vasopressin (AVP) infusion to the system and validated the integrated system in dogs with endotoxin-induced shock.

**Methods:**

Based on the circulatory equilibrium framework, the system directly controls systemic vascular resistance and stressed blood volume by titrating NE and Ringer’s acetate solution (RiA), respectively, thereby restoring AP and CO. AVP is automatically initiated once infusion rate of NE is increased higher than 1 μg·kg⁻^1^·min⁻^1^. The minimally invasive CO monitor estimates CO (CO_es_) by a machine-learning analysis of the peripheral AP contour. In eight anesthetized dogs, the system was implemented, and reference CO (CO_ref_) was measured using an aortic flow probe to calibrate CO_es_ and track reference hemodynamics. Endotoxin shock was induced via intravenous lipopolysaccharide infusions. After the target AP and CO_es_ were set to 70 mmHg and 132 (132–133) mL·min^−1^·kg^−1^, respectively, the system was activated.

**Results:**

The lipopolysaccharide infusion significantly reduced AP from 82 (80–85) to 52 (50–56) mmHg and CO_ref_ from 132 (125–146) to 90 (80–103) mL·min^−1^·kg^−1^, and increased the blood lactate level (LAC) from 21 (21–23) to 36 (32–38) mg·dL^−1^. Once the system was activated, infusions of NE and RiA were automatically initiated. Infusion of AVP initiated during the system control was associated with significant reduction in NE dose while maintaining AP. Within 5 min after system activation, AP and CO_es_ were recovered to >65 mmHg and >122 (122–123) ml·min^−1^·kg^−1^, respectively. The median absolute performance errors for AP and CO_es_ were <5%. At 60 min after system activation, AP and CO_ref_ significantly increased to 72 (68–75) mmHg and 130 (127–137) ml·min^−1^·kg^−1^ and LAC significantly decreased to 26 (25–28) mg·dL^−1^ relative to the values observed at endotoxin shock. CO_es_ tracked directional changes in CO_ref_ reasonably well.

**Conclusions:**

This proof-of-concept study validates the feasibility and physiological performance of the system for the hemodynamic management of vasoplegic shock.

**Supplementary Information:**

The online version contains supplementary material available at https://doi.org/10.1186/s40635-026-00977-3.

## Introduction

Vasoplegic shock is common, contributing up to two-thirds of cases of shock admitted to the intensive care unit [1]. The two most common causes of vasoplegic shock are septic shock and vasoplegic shock seen after cardiopulmonary bypass [2, 3]. In the hemodynamic management of vasoplegic shock, the Surviving Sepsis Campaign (SSC) guidelines or expert opinions recommend appropriate fluid replacement and the use of vasopressors to improve the systemic arterial pressure (AP), systemic perfusion (cardiac output, CO), and blood lactate level (LAC) [2, 3]. Implementation of the SSC guidelines from the early phase of resuscitation is associated with reduced mortality; however, there is still room for improvement in mortality outcomes, even when compliance is high [4]. To improve clinical outcomes, it has been recommended that guideline-based hemodynamic management be tailored to the physiological status of individual patients in a cause-specific manner, for example, adjusting fluid administration according to each patient’s volume status [4]. However, such advanced hemodynamic management is not easily implemented, presumably due to the intrinsic complexity and highly dynamic nature of the shock hemodynamics, as well as the increased workload for intensivists associated with intensive monitoring and frequent titration of drugs and fluids.

A potential solution to these challenges is the automation of the hemodynamic management [5], based on established guidelines and a patient-specific physiological circulatory model. In line with this concept, we previously developed an automated hemodynamic resuscitation system for vasoplegic shock [6, 7]. In endotoxin-induced shock, our previous system continuously evaluated the stressed blood volume and systemic vascular resistance based on the circulatory equilibrium framework [8, 9] and controlled them by automatically titrating the infusion of Ringer’s acetate solution (RiA) and norepinephrine (NE), thereby improving AP and CO to their respective target values. The system provided cause-specific treatment; that is, fluid infusion was in accordance with the blood volume status whereas vasopressor infusion aligned with the degree of vasodilation. Although the system was potentially useful and efficacious in the hemodynamic resuscitation of vasoplegic shock, several issues should be addressed to improve its practical feasibility and efficacy. First, CO monitoring was complex, involving a pre-cordially attached ultrasound Doppler probe [10], which may not be feasible in routine clinical practice. Second, although vasopressin (AVP) infusion is widely used as an adjunct to NE to improve AP and reduce the requirement for NE [3, 11], our previous system lacked AVP infusion control. In vasoplegic shock, relative AVP deficiency contributes to vasodilation and reduced vascular responsiveness to NE [3, 12, 13]. From a pathophysiological standpoint, AVP infusion control should be incorporated into our system.

In our recent developments, we have created a minimally invasive CO monitor that continuously estimates CO using a machine-learning analysis of the peripheral AP contour [14]. Furthermore, we have developed an automated hemodynamic management system incorporating this CO monitor to prevent hypotension and reductions in CO during surgical anesthesia [15]. However, the current system lacks control of AVP infusion and has not yet been validated under hemodynamic conditions relevant to vasoplegic shock. Accordingly, the aim of this study was to develop an automated closed-loop hemodynamic resuscitation system for vasoplegic shock by integrating AVP infusion and the minimally invasive CO monitor, and to validate its control performance in dogs with endotoxin‑induced shock.

## Materials and methods

### Animals

Eight Beagle dogs (Kitayama Labes, Gifu, Japan) with a median body weight of 10.0 kg (interquartile range, 9–11) and a median age of 1.1 years (1.1–1.1) were included in this study. The study protocol was reviewed and approved by the Institutional Animal Care and Use Committee of the National Cerebral and Cardiovascular Center (Approval No. 25036). All experimental procedures were conducted in accordance with the Guidelines for Animal Experiments established by the Japanese Ministry of Health, Labour and Welfare.

### Automated hemodynamic resuscitation system

**Fig. 1:**
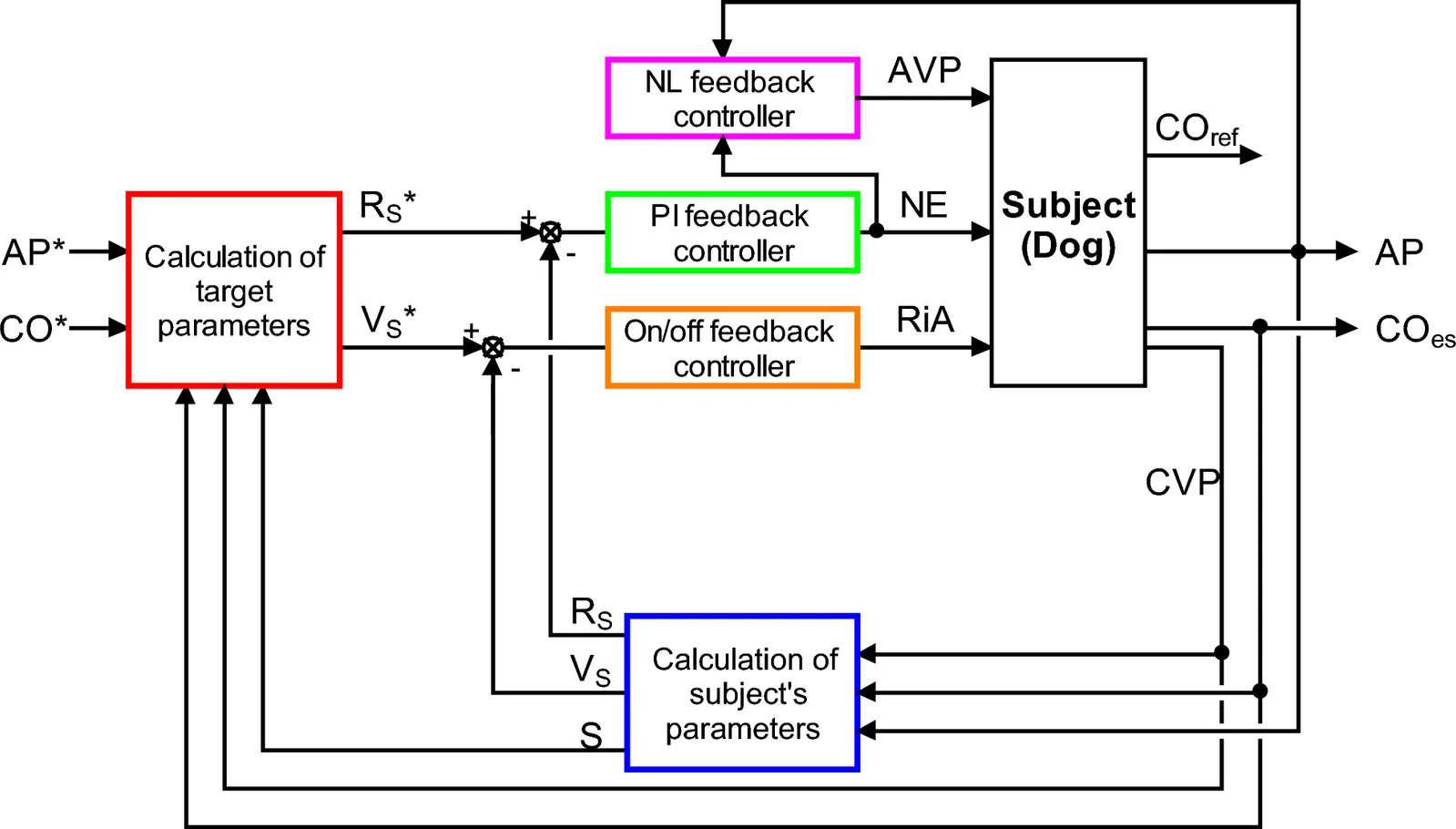
Closed-loop drug infusion system for automated hemodynamic resuscitation in vasoplegic shock. Block diagram of the system for simultaneous control of the mean arterial pressure (AP) and estimated cardiac output (CO_es_) in vasoplegic shock. CO_es_ is estimated by a minimally invasive CO monitor (not shown in the diagram). Systemic vascular resistance (R_S_), systemic stressed blood volume (V_S_), and Frank-Starling slope of the cardiothoracic compartment (S) are calculated from AP, CO_es_, and central venous pressure (CVP) (blue rectangle). AP* and CO* represent target AP and target CO_es_, respectively. From these target variables, the target R_S_ (R_S_*) and V_S_ (V_S_*) are determined (red rectangle). The proportional-integral controller (green rectangle) adjusts the infusion rate of norepinephrine (NE). A non-linear controller (magenta rectangle) adjusts the infusion of vasopressin (AVP). An on/off feedback controller (orange rectangle) adjusts the infusion of Ringer’s acetate solution (RiA)

A schematic illustration of the system developed in this study is provided in Fig. 1. The system was designed to directly control the systemic stressed blood volume (V_S_) and systemic vascular resistance (R_S_) by adjusting the infusion rate of RiA, NE, and AVP using a negative feedback mechanism, thereby controlling AP and estimated CO (CO_es_) (estimated by the minimally invasive CO monitor) to reach the target AP (AP*) and target CO (CO*), respectively. R_S_, V_S_, and slope of the CO curve of the cardiothoracic compartment (S) are continuously calculated from AP, CO_es_, and central venous pressure (CVP) based on the circulatory equilibrium framework [8, 9] (blue rectangle in Fig. 1) (Supplemental Material 1). The system user defines AP* and CO* and inputs them into the system. Based on the AP*, CO*, CO_es_, CVP, and S, the system calculates the target R_S_ (R_S_*) and V_S_ (V_S_*) (red rectangle in Fig. 1). The system repeatedly calculates R_S_* and V_S_* at one-minute intervals and compares them with the continuously calculated R_S_ and V_S_, respectively.

To minimize the difference between R_S_* and R_S_, a proportional-integral feedback controller adjusts the infusion rate of NE (green rectangle in Fig. 1).

To minimize the difference between V_S_* and V_S_, an on/off feedback controller (orange rectangle in Fig. 1) adjusts the RiA infusion rate based on the following “if–then” rule: if V_S_ ≤ V_S_*, then RiA is infused at a rate of 800 mL·h^−1^.

The infusion rate of NE and the on/off state of the RiA infusion are updated every 2 s.

Based on AP and the NE infusion rate, a non-linear feedback controller (magenta rectangle in Fig. 1) adjusts the AVP infusion rate every 5 min based on another “if–then” rules:

If the infusion rate of NE is >1.0 μg·kg^−1^·min^−1^, then infuse AVP at 0.04 IU·min^−1^.

If NE and AVP are concomitantly infused, AP > AP*—5 mmHg, and the infusion rate of NE <0.5 μg·kg^−1^·min^−1^, then reduce the infusion rate of AVP in a stepwise manner.

Further details of the drug controllers are available in Supplemental material 1.

Control processes are executed in parallel within a unified feedback architecture.

### Minimally invasive CO monitor

The minimally invasive CO monitor was originally designed to track the relative CO (%CO) normalized to the baseline CO and to estimate %CO (%CO_es_) using the systolic pressure area in the peripheral AP contour as a surrogate of cardiac stroke volume [14]. %CO_es_ is computed using a machine learning model of the relationship between the reference %CO (a teacher signal), product of the systolic pressure area and heart rate (HR), and mean AP (feature signals), using a support vector regressor [14, 16]. In our previous study [15], we trained the machine-learning model using ten beagle dogs, where %CO and %CO_es_ were tightly correlated (%CO=%CO_es_+0.6, coefficient of determination (R^2^) = 0.72, p<0.001). With the machine-learning model thus trained, the CO monitor was integrated into a notebook PC (CF-FV, Panasonic, Japan) (PC_1). Every 10 s, the CO monitor samples the peripheral AP signal, processes the signal, and outputs %CO_es_ to the system.

To obtain CO_es_ (Fig. 1), %CO_es_ was calibrated against a reference CO (CO_ref_) to derive the absolute CO value (mL·min⁻^1^·kg⁻^1^), which is different from the uncalibrated CO monitor available in clinical practice [17].

### Preparation

**Fig. 2:**
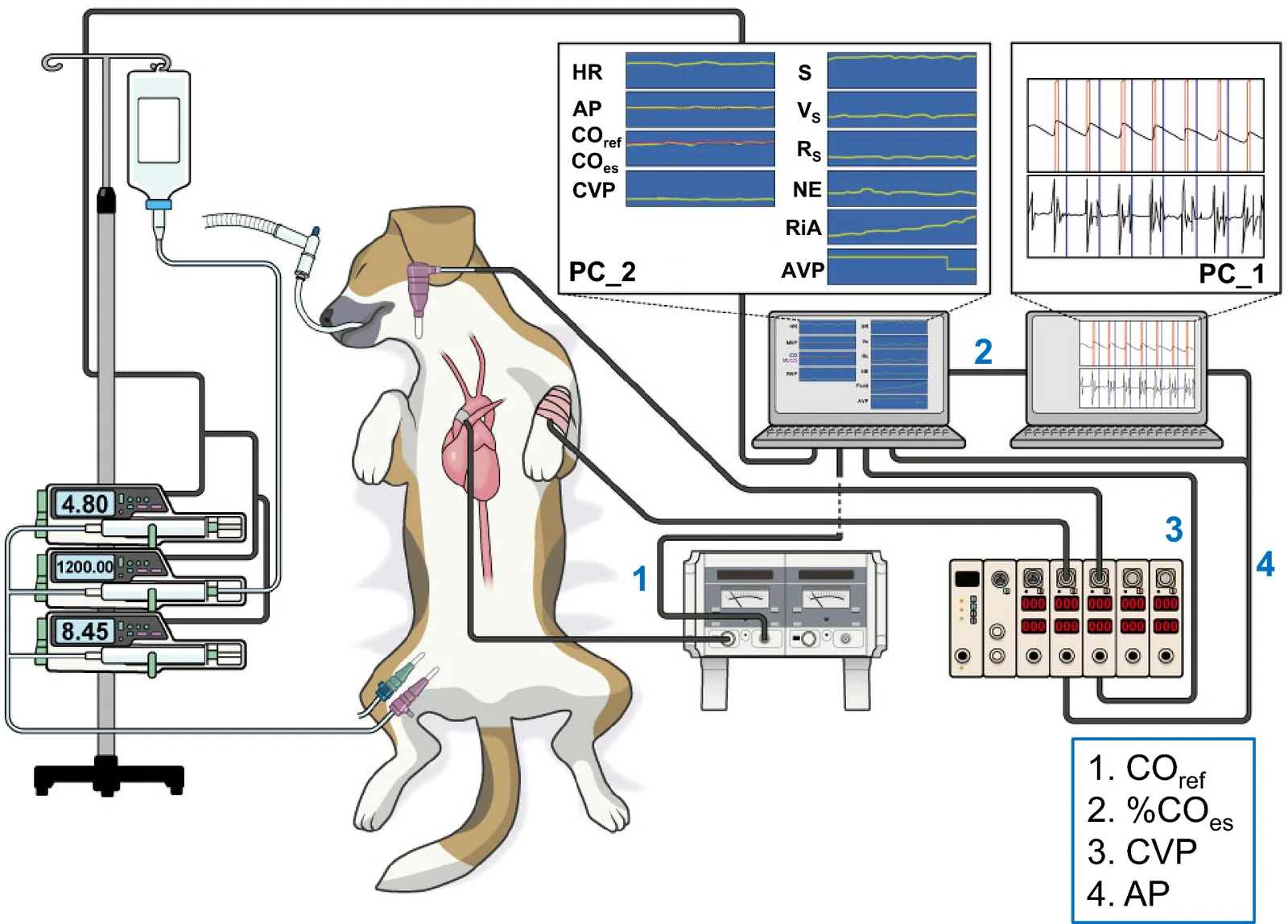
Schematic representation of experimental preparation. Arterial pressure (AP) was measured via a catheter placed in the left radial artery and connected to a pressure transducer (not shown) via a fluid-filled, noncompliant tubing system and biological amplifier. The central venous pressure (CVP) was measured via a catheter placed in the right jugular vein and connected to a pressure transducer (not shown) via a fluid-filled, noncompliant tubing system and biological amplifier. The reference cardiac output (CO_ref_) was measured using an ultrasonic flow probe placed around the ascending aorta. A computer (PC_1) estimated the relative CO (%CO_es_) from the radial arterial pressure contour using a machine learning model. The analog signals of AP, CVP, CO_ref_, and %CO_es_ were transmitted to another laboratory computer (PC_2), which executed the closed-loop control algorithm for the automated infusion of norepinephrine (NE), vasopressin (AVP), and Ringer’s acetate solution (RiA). NE, AVP, and RiA were administered using dedicated infusion pumps connected to the right femoral venous catheter, with infusion rates continuously adjusted by the control algorithm

General anesthesia was induced using intravenous propofol to effect, followed by endotracheal intubation and mechanical ventilation. HR was continuously monitored using surface electrocardiography, and anesthesia was maintained with 1.5–2.0% isoflurane. A pulse oximeter probe (Radical-7, Masimo, USA) was placed on the auricle to measure the peripheral oxygen saturation. A 4-Fr fluid-filled catheter was inserted into the left radial artery and connected to a pressure transducer (DX-200, Nihon Kohden, Japan) to measure AP (Fig. 2). An 8-Fr catheter was placed in the right femoral artery for blood sampling. A 10-Fr catheter was placed in the right femoral vein, through which RiA (Veen F, Fuso, Japan) and rocuronium bromide (Rocuronium Bromide, Maruishi, Japan) were infused at a rate of 4 mL·kg^−1^·h^−1^ and 8 μg·kg^−1^·min^−1^, respectively. A 10-Fr catheter was placed in the right jugular vein, and its side-port was connected to a pressure transducer to measure CVP. A left thoracotomy was performed and an ultrasonic flowmeter (12A594, Transonics Systems Inc., USA) was placed around the ascending aorta to measure CO_ref_ (mL·kg^−1^·min^−1^) (Figs. 1, 2).

NE (Noradrenaline, Alfresa, Japan) was diluted in normal saline to a final concentration of 100 μg·mL^−1^ and loaded into a 50 mL syringe. AVP (Pitressin, Pfizer, Japan) was diluted in normal saline to a concentration of 0.5 IU·mL^−1^ and prepared in a 50 mL syringe. These syringes were then mounted on syringe pumps (TE-SS835N, Terumo, Japan). RiA was administered using an infusion pump (TE-LM835A, Terumo, Japan). The infusion lines from these pumps were connected to the catheter in the right femoral vein (Fig. 2), and the pumps were controlled by a laboratory computer (LC-72 N10, Logitec, Japan) (PC_2).

### Data acquisition, signal processing, and command generation

The analog AP signal was input into PC_1 to compute %CO_es_ (%) (Fig. 2). The analog signals of AP, CVP, %CO_es_, and CO_ref_ were digitized (200 Hz, 12-bit) by PC_2 and smoothed using a low-pass filter (time constant 10 s) to reduce noise and stabilize control performance of the system. Every 2 s, the control program in PC_2 computed the infusion rates of NE, AVP, and RiA, and transmitted command signals to the infusion pumps.

### Protocols

Following the completion of all preparatory procedures, a 30 min stabilization period was allowed, and baseline hemodynamic data (Base) were collected. Endotoxin shock was induced via the intravenous administration of *Escherichia coli* lipopolysaccharide (LPS; 055:B5, Sigma Laboratories, St. Louis, MO) prepared at a concentration of 10 mg·mL⁻^1^ in saline. Each animal received a total dose of 4–10 mg·kg⁻^1^ of LPS infused via the right femoral vein catheter over a period of 60 min. The LPS dose was adjusted such that AP was reduced to <60 mmHg. At 60 min after initiating the induction of endotoxin shock, hemodynamic data were collected (Shock).

During the pre-control period of 5 min, %CO_es_ was calibrated once to CO_ref_ to determine CO_es_. We defined AP* as 70 mmHg in all animals, since the SSC guidelines recommend that the AP is maintained at >65 mmHg [2]. If CO_ref_ were to be a controlled variable, CO* should have been 120 mL·min^−1^·kg^−1^ [6], which corresponds to a cardiac index of 2.5 L·min^−1^·m^−2^ in 10 kg bodyweight dogs. However, since CO_es_ was used as a system-controlled variable, CO* for CO_es_ was defined as 132 (132–133) mL·min^−1^·kg^−1^, considering the estimation error of the minimally invasive CO monitor [14, 15]. The system was activated by closing the feedback loops (Fig. 1). The infusion rates of the drugs were continuously recorded in PC_2. System performance was observed for 60 min following activation. Hemodynamic data at the end of this observation period (Resuscitated) were collected. After deactivating the system at 60 min, we observed the hemodynamics for 5 min (post-control period).

LAC was measured at the three time points: Base, Shock, and Resuscitated, using an analyzer (GEM Premier 5000, Instrumentation Laboratory, USA).

### Statistical data analysis

Group data are presented as medians with interquartile ranges because preliminary analyses using the Shapiro–Wilk test demonstrated that several variables significantly deviated from a normal distribution. No data transformations, such as logarithmic transformation, were performed to improve data normality. A two-sided P value of <0.05 was considered statistically significant. Statistical analyses were conducted using commercially available software (GraphPad Prism 11, GraphPad Software, Inc., USA) or custom-made software coded with Python.

### Reference hemodynamic and LAC data at base, shock, and resuscitated

We calculated the reference R_S_ (R_Sref_), V_S_ (V_Sref_), and S (S_ref_) using the CO_ref_ in a similar manner as that used for R_S_, V_S_, and S calculation with use of the CO_es_ (Supplemental Material 1). HR, AP, CO_ref_, CVP, R_Sref_, V_Sref_, and S_ref_ were averaged over 10 s at Base, Shock, and Resuscitated. Overall differences among the three time points were assessed using the Friedman test. When a significant overall effect was detected, prespecified pairwise comparisons between adjacent time points were performed using the Wilcoxon signed-rank test with Bonferroni correction for multiple comparisons [6, 7].

### Control performance of the system

To evaluate the rapidity of the system control, we calculated the response time required for AP and CO_es_ to reach respective acceptable ranges, which were defined as 65 mmHg for AP, and CO* − 10 mL·min^−1^·kg^−1^ for the CO_es_. To assess the accuracy of hemodynamic control achieved by the system, performance error metrics were evaluated as previously described [6, 18]. For each animal, the following indices were calculated for the AP and CO_es_ recorded from 10 to 60 min after system activation: percentage error (PE), defined as the difference between the measured value and the corresponding target value, expressed as a percentage of the target; median performance error (MDPE), representing the median of all PE values; median absolute performance error (MDAPE), defined as the median of the absolute PE value; wobble, reflecting the variability of PE around the MDPE and calculated as the median absolute deviation of PE from MDPE; and divergence, representing the temporal trend in the absolute performance error.

### Effect of AVP infusion on the NE infusion rate and hemodynamics

The AVP and NE infusion rates as well as AP and CO_es_ were determined at the time of AVP initiation and 20 min after its initiation. Variables between the two time points were compared using the Wilcoxon signed-rank test.

### Performance of the minimally invasive CO monitor

CO_ref_ and CO_es_ measurements collected during the 70-min experimental protocol (5 min pre-control, 60 min system control, and 5 min post-control) were averaged every 10 s, resulting in 420 data sets per animal and 3,360 data sets in total across the eight animals. The association between the CO_ref_ and CO_es_ was evaluated using Spearman’s rank correlation coefficient. We analyzed the agreement between CO_ref_ and CO_es_ with a modified method of Bland and Altman for repeated measurements within animals [16, 19]. We calculated bias as the mean of the differences (= CO_es_—CO_ref_), the limit of agreement (LOA = bias ± 1.96 × standard deviation of the differences) [16], 95% confidence intervals of the LOA by bootstrap resampling (2,000 iterations), and percentage error (1.96 × [standard deviation of the differences]/ [mean CO] × 100). Two methods of measuring CO are judged to be interchangeable if LOA did not exceed 20% of the mean CO [20]. We assessed the association between the difference and the mean using Spearman’s rank correlation coefficient. We analyzed the trending ability of relative changes in CO using the 4 quadrant plot method [21, 22]. The percentage changes in CO_ref_ (%ΔCO_ref_) and CO_es_ (%ΔCO_es_) were calculated from consecutive measurements obtained 1 min apart. Using a central exclusion zone of 15% [22], we calculated the concordance rate between %ΔCO_ref_ and %ΔCO_es_.

## Results

**Fig. 3:**
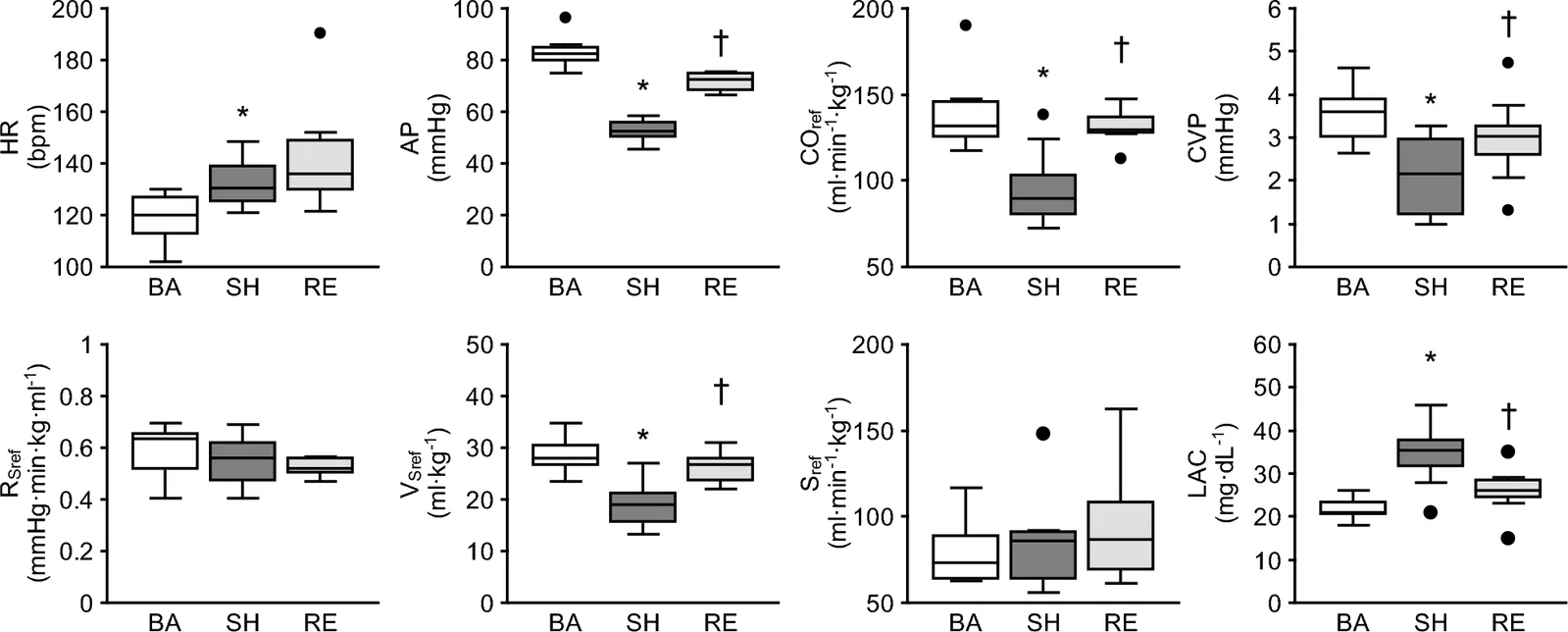
Hemodynamic variables and blood lactate levels during endotoxin-induced shock and automated resuscitation. BA, Base; SH, Shock; RE, Resuscitated; HR, heart rate; AP, arterial pressure; CO_ref_, reference cardiac output; CVP, central venous pressure; R_Sref_, reference systemic vascular resistance; V_Sref_, reference systemic stressed blood volume; S_ref_, reference Frank-Starling slope of the CO curve of the cardiothoracic compartment; LAC, blood lactate level. Data are expressed as box plots, displaying the median (line inside box), 1st and 3rd quartiles (lower and upper ends of the box), range (lower and upper ends of whiskers), and outliers if any. *P<0.05 vs BA. ^†^*P*<0.05 vs SH. (n=8 for each time point)

Data from all eight animals were included in the final analysis. The reference hemodynamic and LAC data during the experiments are summarized in Fig. 3.

### Induction of endotoxin shock

The intravenous administration of LPS successfully induced endotoxin shock in all eight animals (Base vs. Shock, Fig. 3). The induction of endotoxin shock significantly increased the HR and LAC level, and significantly decreased the AP, CO_ref_, CVP, and V_Sref_. There were no significant differences in R_Sref_ and S_ref_ between Base and Shock.

### Hemodynamic resuscitation by the system

**Fig. 4:**
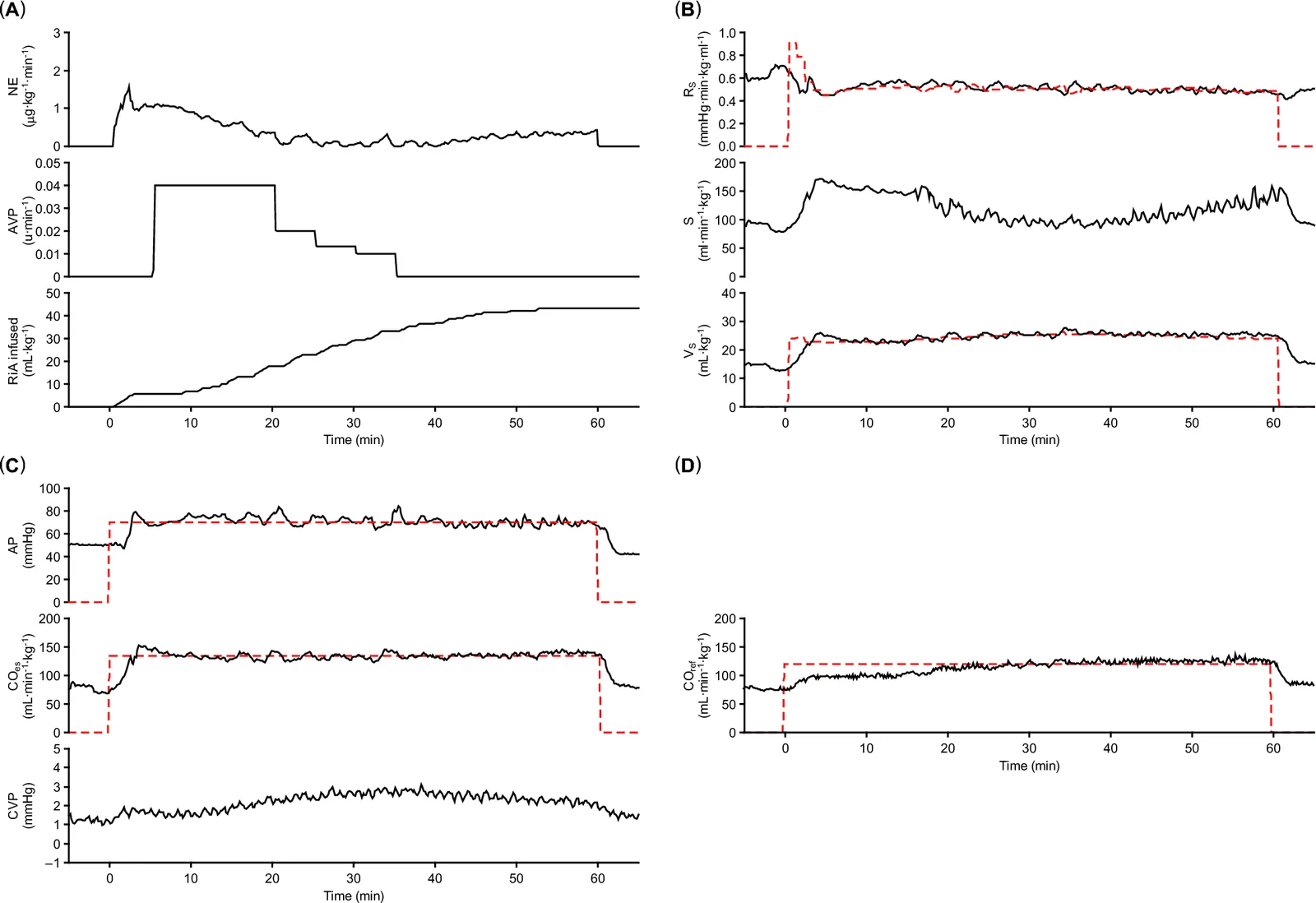
Experimental time course of automated hemodynamic resuscitation in one representative dog over a 60 min period. (**A**) Time courses of norepinephrine (NE) and vasopressin (AVP) infusion rates and cumulated volume of Ringer’s acetate solution (RiA) infused. (**B**) Time courses of systemic vascular resistance (R_S_), Frank-Starling slope of the cardiothoracic compartment (S), and systemic stressed blood volume (V_S_). Red dotted lines indicate the target parameters [top; target R_S_ (R_S_*), bottom; target V_S_ (V_S_*)]. (**C**) Time courses of the mean arterial pressure (AP), estimated cardiac output (CO_es_), and central venous pressure (CVP). Red dotted lines indicate the target hemodynamic variables [top; target AP (AP*), second from the top; target CO_es_ (CO*)]. (**D**) Time course of reference CO (CO_ref_). The AP* and CO* were constant throughout the control period, while the R* and V* were recalculated every minute

**Fig. 5:**
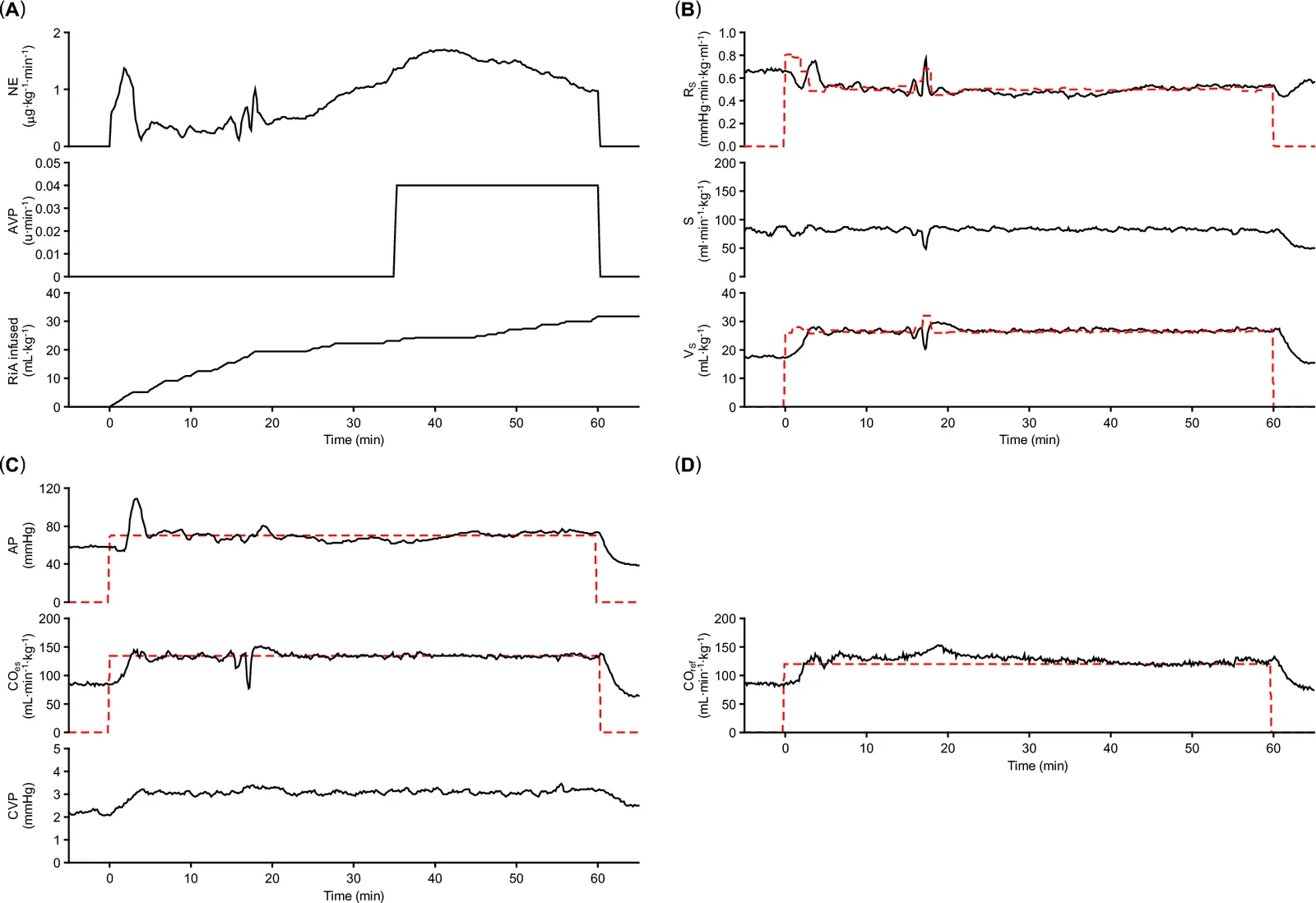
Experimental time course of automated hemodynamic resuscitation in another representative dog over a 60 min period. (**A**) Time courses of norepinephrine (NE) and vasopressin (AVP) infusion rates and cumulated volume of Ringer’s acetate solution (RiA) infused. (**B**) Time courses of systemic vascular resistance (R_S_), Frank-Starling slope of the cardiothoracic compartment (S), and systemic stressed blood volume (V_S_). Red dotted lines indicate the target parameters [top; target R_S_ (R_S_*), bottom; target V_S_ (V_S_*)]. (**C**) Time courses of the mean arterial pressure (AP), estimated cardiac output (CO_es_), and central venous pressure (CVP). Red dotted lines indicate the target hemodynamic variables [top; target AP (AP*), second from the top; target CO_es_ (CO*)]. (**D**) Time course of reference CO (CO_ref_). The AP* and CO* were constant throughout the control period, while the R* and V* were recalculated every minute

Figure 4 shows the experimental data from one animal. Once the system was activated at 0 min, infusion of NE and RiA were initiated (Fig. 4A). At 5 min after system activation, since the infusion rate of NE was >1 *μ *g·kg^−1^·min^−1^, AVP infusion was initiated. These controls of NE, AVP, and RiA promptly improved R_S_ and V_S_ to their respective target values (red dotted lines in Fig. 4B). Following the control of R_S_ and V_S_, AP and CO_es_ were rapidly and accurately controlled to their respective target values (red dotted lines in Fig. 4C). CO_ref_ was also recovered to ~120 mL·min^−1^·kg^−1^ at approximately 20 min after system activation. The response times for AP and CO_es_ were 2.8 and 2.3 min, respectively. MDAPEs for AP and CO_es_ were <4%. Figure 5 shows the experimental data for another animal. Once the system was activated at 0 min, infusions of NE and RiA were initiated (Fig. 5A). These controls of NE and RiA promptly improved R_S_, V_S_, AP and CO_es_ to their respective target values (red dotted lines in Fig. 5B and C). AVP infusion was initiated at 35 min after system activation since the infusion rate of NE was gradually increased to >1 μg·kg^−1^·min^−1^. CO_ref_ was promptly recovered to 120 mL·min^−1^·kg^−1^ at approximately 5 min after system activation. The response times for AP and CO_es_ were 2.8 and 3.7 min, respectively. MDAPEs for AP and CO_es_ were <5%. Interestingly, in both animals, after the initiation of AVP infusion, the infusion rate of NE was automatically and gradually reduced, while AP was maintained to AP* precisely. Once the system was deactivated at 60 min, AP, CO_es_ and CO_ref_ abruptly decreased in both animals, supporting the efficacy of hemodynamic resuscitation by the system. Data traces obtained in the remaining six animals are provided in Supplemental Material 2.

**Fig. 6:**
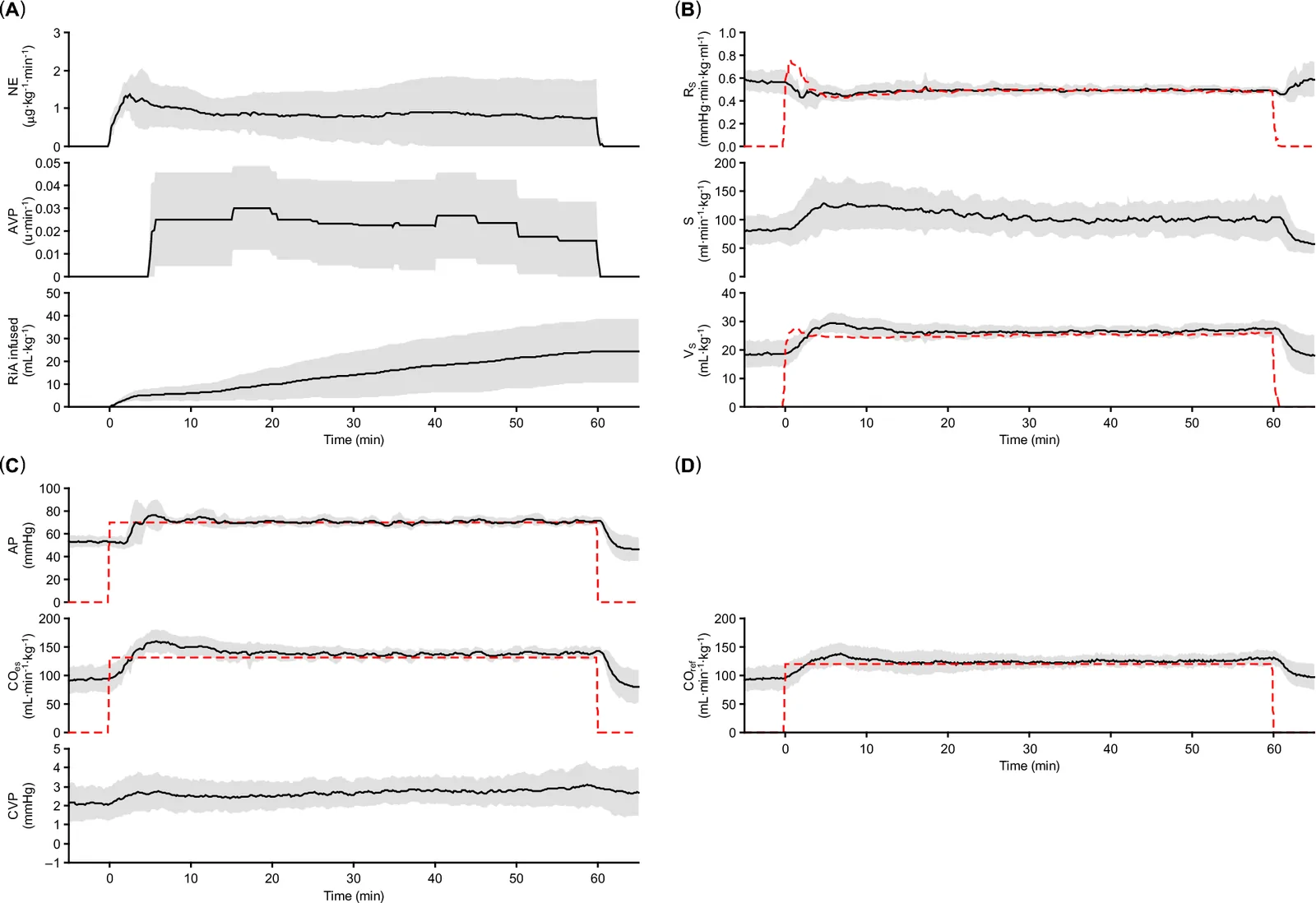
Summarized experimental time course of automated hemodynamic resuscitation in all eight dogs over a 60 min period. Data are expressed as the median (solid line) and interquartile range (gray area). (**A**) Time courses of norepinephrine (NE) and vasopressin (AVP) infusion rates and cumulated volume of Ringer’s acetate solution (RiA) infused. (**B**) Time courses of systemic vascular resistance (R_S_), Frank-Starling slope of the cardiothoracic compartment (S), and systemic stressed blood volume (V_S_). Red dotted lines indicate the median target parameters [top; target R_S_ (R_S_*), bottom; target V_S_ (V_S_*)]. (**C**) Time courses of the mean arterial pressure (AP), estimated cardiac output (CO_es_), and central venous pressure (CVP). Red dotted lines indicate the median target hemodynamic variables [top; target AP (AP*), second from the top; target CO_es_ (CO*)]. (**D**) Time course of reference CO (CO_ref_). The AP* and CO* were constant throughout the control period, while the R* and V* were recalculated every minute

Figure 6 summarizes the hemodynamic responses during system control in all animals. As shown in Fig. 6A, NE and RiA infusions were initiated immediately upon system activation at 0 min in all animals. In five of the eight (62.5%) animals, AVP infusion was initiated at 5 min after system activation. The time-averaged infusion rates over the 60 min control period were 0.64 (0.60–0.86) μg·kg^−1^·min^−1^ for NE and 0.02 (0.01–0.03) IU·min⁻^1^ for AVP. The cumulative volume of RiA administered was 26 (14–34) ml·kg⁻^1^. As shown in Fig. 6B, after system activation, R_S_ initially decreased to varying degrees across animals, followed by gradual convergence toward the target value, while V_S_ progressively approached its target level. As illustrated in Fig. 6C, AP and CO_es_ were recovered rapidly and remained well-controlled at their respective targets in all animals. Although not directly controlled, CO_ref_ was also recovered at ~120 mL·min^−1^·kg^−1^ (Fig. 6D). As summarized in Table 1, AVP infusion was associated with a significant reduction in the NE infusion rate at 20 min after AVP initiation while maintaining AP. CO_es_ also showed a significant decrease following AVP administration; however, this reflects the process of converging toward the CO*. The control performance of AP and CO_es_ over the eight animals is summarized in Table 2. The median response times were <5 min for the AP and CO_es_, indicating the rapidity of the system control, considering that the SSC guidelines recommend hemodynamic optimization within the 1 h bundle. The MDAPEs for AP and CO_es_ were <5%, indicating precise system control. Small wobble and divergence values also indicate the stability of the system control. Once the system was deactivated at 60 min, the hemodynamics collapsed, substantiating the efficacy of the automated resuscitation system.

**Table 1:** Changes in hemodynamics and vasopressor doses after initiation of vasopressin infusion

	**Initiation of AVP infusion**	**20 min after initiation of AVP infusion**
AP (mmHg)	70 (66–72)	72 (69–75)
CO_es_ (ml·min^−1^·kg^−1^)	155 (146–166)	136 (131–141)*
Infusion rate of AVP (IU·min⁻^1^)	0.04 (0.04–0.04)	0.04 (0.01–0.04)
Infusion rate of NE (μg·kg⁻^1^·min⁻^1^)	1.33 (1.08–1.45)	0.69 (0.17–0.87)*

Values are presented as the median (interquartile range). AP, arterial pressure; CO_es_, cardiac output estimated by the minimally invasive CO monitor; AVP, vasopressin; NE, norepinephrine. (n=8 for each variable) *P<0.05 vs Initiation of AVP infusion

**Table 2:** Performance of system control of arterial pressure and cardiac output

	**Response time (min)**	**MDPE** **(%)**	**MDAPE** **(%)**	**Wobble** **(%)**	**Divergence** **(%·min**^**−1**^**)**
AP	3.8 (3.1–4.7)	0.7 (0.2–1.9)	4.0 (3.3–5.1)	3.7 (2.5–4.8)	0.00 (0.00–0.00)
CO_es_	2.6 (1.7–3.2)	3.3 (1.8–5.0)	3.5 (2.9–6.9)	2.7 (2.3–3.7)	0.00 (0.00–0.00)

Values are presented as the median (interquartile range). AP, arterial pressure; CO_es_, cardiac output estimated by the minimally invasive CO monitor; MDPE, median performance error; MDAPE, median absolute performance error. (n=8 for each variable)

Hemodynamic resuscitation by the system significantly increased AP, CO_ref_, CVP, and V_Sref_, and significantly decreased LAC (Shock vs. Resuscitated, Fig. 3).

### Reliability of the minimally invasive CO monitor

**Fig. 7:**
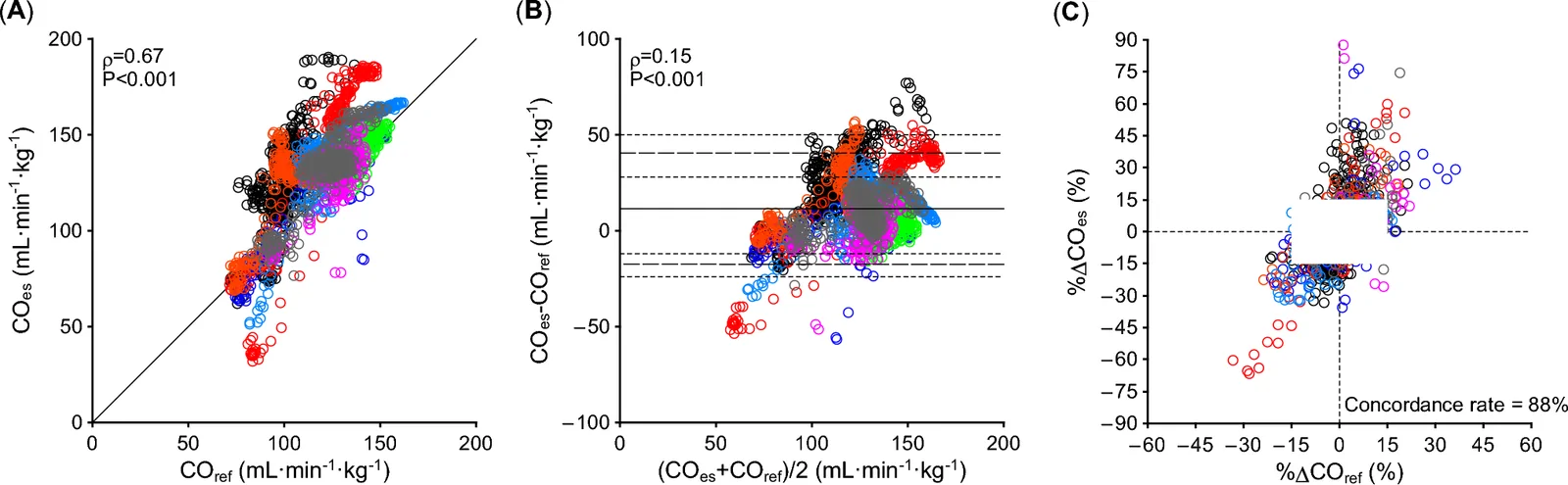
Relation between the estimated and reference cardiac output during the 70 min period. (**A**) Correlation between the reference cardiac output (CO_ref_) and estimated cardiac output (CO_es_). (**B**) Bland–Altman analysis between CO_ref_ and CO_es_. (**C**) Four quadrant plot analysis to evaluate concordance rate between relative changes in CO_ref_ (%ΔCO_ref_) and CO_es_ (%ΔCO_es_) with an exclusion zone of 15%. The individual animals are represented by different colors. In (**A**) and (**B**), Spearman’s rank correlation coefficient (ρ) and probability value are shown. In (**A**), the thin solid line represents the line of equality. In (**B**), the thin solid line, the thin dashed lines, and the thin dotted lines represent the mean difference (bias), the limits of agreement (LOA=bias ± 1.96 standard deviation), and the 95% confidence interval of LOA, respectively

As shown in Fig. 7A, CO_es_ and CO_ref_ obtained during the experiment were moderately correlated with Spearman’s rank correlation coefficient of 0.67. As shown in Fig. 7B, Bland–Altman analysis indicated that the bias was 12 mL·kg^−1^·min^−1^, and the LOA ranged from − 18 to 41 mL·kg^−1^·min^−1^, which corresponded to − 14 to 33% of mean CO (127 mL·kg^−1^·min^−1^). The percentage error was 23%. The differences and the mean values were significantly and positively correlated in the Bland–Altman plot with Spearman’s rank correlation coefficient of 0.15 (Fig. 7B), indicating that the agreement depended on the CO level. In light of the conventional criteria [20, 21], the absolute accuracy of the minimally invasive CO monitor was not sufficient. As shown in Fig. 7C, the 4 quadrant analysis indicated that the concordance rate between %ΔCO_ref_ and %ΔCO_es_ was 88%, indicating that the CO monitor tracked the directional changes in CO reasonably well [22].

## Discussion

In this study, we developed and validated a novel automated hemodynamic resuscitation system for computer-controlled dual vasopressors and fluid therapy in the management of endotoxin-induced shock. The principal findings are threefold: first, the system rapidly, precisely, and stably restored and maintained AP and CO at predefined targets under shock conditions. Second, the integration of AVP infusion expanded the system control domain beyond catecholamine-dependent regulation. As far as we know, no system has simultaneously controlled NE and AVP, making this approach highly novel. Third, CO estimation derived from the peripheral AP contour enabled the implementation of a less invasive monitoring strategy without compromising control performance. Although the system control was stable in this acute study, this does not guarantee comparable performance throughout the hours-to-days clinical course of human vasoplegic shock [1]. Therefore, further research and development are required before the system can be reliably translated into clinical practice.

The circulatory equilibrium framework decomposes hemodynamics into interpretable components such as R_S_, V_S_ and S. Leveraging this framework, the system enables cause-oriented control rather than apparent regulations of AP and CO [15]. This approach would be particularly efficacious in vasoplegic shock, wherein vasodilation and relative hypovolemia coexist and evolve over time [1, 23]. In this endotoxin shock model, although V_S_ decreased significantly (venous dilation), R_S_ did not (Fig. 3). This is compatible with previous findings in dogs with endotoxin shock [24]. Our system is designed to improve AP as rapidly as possible (Supplementary Material 1), which resulted in the simultaneous initiation of NE and RiA in this endotoxin shock model. This approach is supported by recent clinical evidence [25] indicating that early initiation of NE infusion improves outcomes in patients with septic shock and does not necessarily conflict with the SSC guidelines [2]. During system control, S was continuously estimated to determine the target values of R_S_ and V_S_ (Fig. 1) but was not actively controlled. S is theoretically related to cardiac contractility (end-systolic ventricular elastance), HR, and R_S_ [26]. Based on the observed changes in S, HR, and R_S_ (Fig. 3), a decline in cardiac contractility in this endotoxin shock model appears unlikely. This finding is consistent with a previous report [27]. In contrast, myocardial depression is frequently observed in septic shock [2]. To extend the applicability of the current system to septic shock conditions, it will be necessary to control S through inotropic support (e.g., dobutamine) [28] and to rigorously validate the system in disease models closely replicating septic shock over prolonged periods (at least 1–2 days) [29].

AVP infusion by the system was associated with significant reduction in the infusion rate of NE while maintaining AP (Table 1). Although the optimal timing of AVP initiation remains uncertain, earlier initiation of AVP may be associated with improved outcomes [11, 13]. The SSC guidelines recommend that AVP infusion is started when the infusion rate of NE is increased to 0.25–0.5 μg·kg^−1^·min^−1^ [2, 13], whereas the NE threshold in our system was defined as 1 μg·kg^−1^·min^−1^. This higher threshold is chosen because previous studies using canine endotoxin shock models [6, 7] demonstrated that substantially higher doses of NE were required to achieve adequate hemodynamic resuscitation. Despite this relatively high NE threshold, our system typically initiates AVP infusion during the very early phase of hemodynamic resuscitation. This may be due to the difference in cardiovascular sensitivity to NE between humans and dogs [30, 31], and the setting of the NE controller in our system. The initial AVP infusion rate used in this system, 0.04 IU·min^−1^, was defined in accordance with a previous canine study [31]. However, a fixed dose of 0.03–0.06 IU·min^−1^ of AVP is recommended in human clinical practice. Considering the body weight of the dogs (10 kg), the initial dose in this system may appear excessive; however, it has been suggested that the effects of AVP are independent of body mass index [13]. The AVP dose can be reduced in a stepwise manner in the system control after hemodynamic recovery. Furthermore, the NE threshold, the initial AVP dose, and the stepwise AVP dose-reduction scheme are all adjustable within the control system. Since the adjunctive use of AVP with NE carries a risk of peripheral ischemia [32], further studies are warranted to determine whether adequate hemodynamic resuscitation can be achieved with an initial AVP infusion rate lower than those used in the present system setting. In any way, the system enables the dynamic administration of AVP based on real-time hemodynamic conditions, shifting the role of AVP from a static adjunct to a continuously adjustable one.

Another important advancement from our previous resuscitation system is the integration of the minimally invasive CO monitor. In our previous system [6], CO monitoring required a bulky ultrasound Dopper probe attached to the chest wall [10], thereby limiting its clinical applicability. With the minimally invasive CO monitor [14], the system control was effective. However, based on the conventional criteria, the absolute accuracy of the minimally invasive CO monitor used in this system is not sufficient (Fig. 7A, B) and this warrants further investigation on its impact on hemodynamic control. When CO_es_ underestimates CO_ref_, V_S_ calculated from CO_es_ and CVP is underestimated, whereas R_S_ calculated from AP, CO_es_, and CVP is overestimated. As a consequence, administration of RiA tends to be excessive despite adequate actual V_S_ (V_Sref_), and NE dosing tends to be suppressed despite a relatively low true R_S_ (R_Sref_). This fluid-dominant resuscitation may improve AP and CO, but at the cost of an increased risk of systemic edema and pulmonary congestion. Conversely, when CO_es_ overestimates CO_ref_, V_S_ is overestimated and R_S_ is underestimated. In this scenario, RiA administration becomes insufficient despite inadequate true V_S_ (V_Sref_), while NE dosing tends to increase despite relatively high true R_S_ (R_Sref_). As a result, NE-dominant resuscitation may be achieved; however, peripheral perfusion may remain suboptimal, with a potential risk of elevated LAC. Indeed, as shown in Fig. 7B, CO_es_ appeared to be accurate at approximately 100 mL·min⁻^1^·kg⁻^1^, but it underestimated CO_ref_ at lower values and overestimated CO_ref_ at higher values, underscoring the potential risks described above. However, after the automated resuscitation, CVP did not reach abnormally high levels, and LAC improved, suggesting that the impact of these risks was limited in the present validation study. One possible explanation is that the degree of underestimation or overestimation was not large enough to increase the associated risks. The minimally invasive CO monitor tracks directional changes in CO reasonably well (Fig. 7C). This may be discrepant with the moderate correlation observed between CO_ref_ and CO_es_, but probably due to the fact that a total of 3,054 out of 3,360 data sets (90.9%) were excluded by the 15% exclusion zone, in which small variations in CO could hamper the strength of correlation. This high exclusion rate was attributable to the closed-loop control system maintaining CO_es_ accurately near the target value, resulting in only small changes in CO (%ΔCO < 15%) during most of the study period as evidenced in Fig. 6. Therefore, the concordance rate of 88% primarily reflects the ability of the monitor to track relatively large changes in CO rather than its performance during stable control periods. Taken together, even with such degree of absolute agreement with true CO signal (CO_ref_), the minimally invasive CO monitor may enable acceptable hemodynamic resuscitation if the CO_es_ provided by the monitor tracks directional changes in CO reasonably well and the target CO (CO*) is determined with due consideration of the limits in accuracy. Because the conventional criteria are conventional rather than evidence-based thresholds, the utility of a CO monitor may not necessarily be determined solely by adherence to these criteria. Nevertheless, when using CO_es_, the potential risks should always be considered in system operation. At the same time, further improvements in the accuracy of CO_es_ remain essential.

Several minimally invasive CO monitors based on peripheral AP contour analysis are also available for clinical use [33] and may be integrated into our system in future clinical applications; however, concerns regarding their reliability during septic shock remain. In any way, the SSC guidelines do not necessarily recommend the use of minimally invasive CO monitors in the hemodynamic management [2]. This may stem from the insufficient utilization of the CO values obtained from the monitor in clinical management. Visualizing CO‑derived parameters, such as the V_S_ and R_S_, and utilizing them in cause-specific hemodynamic management may redefine the clinical value of the minimally invasive CO monitor. Further research on this issue is warranted.

The LAC was not specifically considered in the automated hemodynamic resuscitation system. Fortunately, the LACs were significantly reduced after automated resuscitation. This suggests that the definition of the CO* in our protocol is appropriate in terms of the optimization of the LACs. The SSC guidelines do not recommend a fixed CO* value [2]. Taken together, dynamically adjusting the CO* based on the current LAC or microcirculation dynamics [34] may be more appropriate for an effective treatment of vasoplegic shock. Furthermore, it may be necessary to tailor AP* to the individual subject’s condition, once the hemodynamic condition stabilizes.

The system may reduce the stress and workload of the intensivists and associated care providers and relocate their efforts to other important activities including respiratory management and infection control, as the system merely requires supervision during the automated resuscitation, eliminating the need for intensive bedside hemodynamic monitoring and repetitive manual drug titration by the clinicians.

Several limitations of this study should be acknowledged. The endotoxin-induced shock model used in this study should be regarded as an experimental model of the vasoplegic shock. MQTiPSS guidelines indicate that LPS administration does not replicate human septic shock [29] and human septic shock is frequently complicated by multiple organ failure including cardiac dysfunction [2]. The young and small-sized beagle dogs free from co-morbidities used in this study are not an accurate representation of clinical patients with vasoplegic shock, who are typically elderly and have several co-morbidities such as renal disorders. The system was evaluated for only 60 min, and its long-term performance was not assessed. We did not compare the efficacy of the automated hemodynamic resuscitation to that of manual resuscitation as in our previous study [15]. The drug controller settings in this system (Fig. 1) and the machine-learning model in the CO monitor were developed using canine data; however, they have not yet been adjusted for humans. Furthermore, adaptive tuning of the drug controller settings for individual patients would be desirable in future clinical applications [35]. Although the drug controllers used in this study were tuned based on population-averaged drug responses (Supplementary Materials 1), minor oscillations in AP and the NE infusion rate were observed in one animal (Supplementary Materials 2; Animals #1). In the present study, CVP was not abnormally elevated; however, fluid overload could not be entirely ruled out. Objective measures of extravascular lung water or pulmonary edema such as the ratio of the arterial partial pressure of oxygen to the inspired oxygen fraction should have been monitored in this study. These issues should be addressed in future research and developments.

In conclusion, we developed an integrated closed-loop system for automated hemodynamic resuscitation that incorporates AVP control and minimally invasive CO monitoring. Building upon prior systems and recent advancements, this approach enables the continuous, cause-specific control of hemodynamic resuscitation and may contribute to the development of personalized and automated resuscitation strategies in the management of vasoplegic shock.

## Supplementary Information

Supplementary Material 1.

Supplementary Material 2.

## Data Availability

Additional data are available from the corresponding author upon reasonable request.

## Abbreviations

SSC: Surviving sepsis campaign
AP: Arterial pressure
CO: Cardiac output
LAC: Lactate level
RiA: Ringer’s acetate solution
NE: Norepinephrine
AVP: Vasopressin
V_S_: Systemic stressed blood volume
R_S_: Systemic vascular resistance
AP*: Target AP
CO*: Target CO
CO_es_: Estimated CO
CO_ref_: Reference CO
CVP: Central venous pressure
S: Slope of the CO curve of the cardiothoracic compartment
R_S_*: Target R_S_
V_S_*: Target V_S_
PI: Proportional-integral
K_i_: Integral gain
K_p_: Proportional gain
%CO: Relative CO
%CO_es_: Estimated %CO
HR: Heart rate
R^2^: Coefficient of determination
Base: Baseline hemodynamic data
LPS: Lipopolysaccharide
Shock: Hemodynamic data at 60 min after endotoxin shock induction
Resuscitated: Hemodynamic data at the end of the observation period
R_Sref_: Reference R_S_
V_Sref_: Reference V_S_
S_ref_: Reference S
PE: Percentage error
MDPE: Median performance error
MDAPE: Median absolute performance error
LOA: Limit of agreement

## References

[1] Mistry RN, Winearls JE (2025) Management of vasoplegic shock. BJA Educ 25(2):65–73. 10.1016/j.bjae.2024.10.004 39897429 PMC11785552

[2] Prescott HC, Antonelli M, Alhazzani W et al (2026) Surviving sepsis campaign: international guidelines for management of sepsis and septic shock 2026. Crit Care Med 54(4):725–812. 10.1097/CCM.0000000000007075 41869847

[3] Busse LW, Barker N, Petersen C (2020) Vasoplegic syndrome following cardiothoracic surgery-review of pathophysiology and update of treatment options. Crit Care 24(1):36. 10.1186/s13054-020-2743-8 32019600 PMC7001322

[4] De Backer D, Cecconi M, Chew MS et al (2022) A plea for personalization of the hemodynamic management of septic shock. Crit Care 26(1):372. 10.1186/s13054-022-04255-y 36457089 PMC9714237

[5] Merouani M, Guignard B, Vincent F et al (2008) Norepinephrine weaning in septic shock patients by closed loop control based on fuzzy logic. Crit Care 12(6):R155. 10.1186/cc7149 19068113 PMC2646320

[6] Uemura K, Kawada T, Zheng C, Li M, Sugimachi M (2017) Computer-controlled closed-loop drug infusion system for automated hemodynamic resuscitation in endotoxin-induced shock. BMC Anesthesiol 17(1):145. 10.1186/s12871-017-0437-9 29061119 PMC5654105

[7] Uemura K, Kawada T, Zheng C, Li M, Sugimachi M (2019) Low-dose landiolol reduces heart rate and cardiac oxygen consumption without compromising initial hemodynamic resuscitation in a canine model of endotoxin shock. Shock 52(1):102–110. 10.1097/SHK.0000000000001224 30052577

[8] Guyton AC (1955) Determination of cardiac output by equating venous return curves with cardiac response curves. Physiol Rev 35(1):123–129. 10.1152/physrev.1955.35.1.123 14356924

[9] Uemura K, Sugimachi M, Kawada T et al (2004) A novel framework of circulatory equilibrium. Am J Physiol Heart Circ Physiol 286(6):H2376-2385. 10.1152/ajpheart.00654.2003 14764438

[10] Uemura K, Kawada T, Inagaki M, Sugimachi M (2013) A minimally invasive monitoring system of cardiac output using aortic flow velocity and peripheral arterial pressure profile. Anesth Analg 116(5):1006–1017. 10.1213/ANE.0b013e31828a75bd 23492964

[11] Kalimouttou A, Kennedy JN, Feng J et al (2025) Optimal vasopressin initiation in septic shock: the OVISS reinforcement learning study. JAMA 333(19):1688–1698. 10.1001/jama.2025.3046 40098600 PMC11920879

[12] Russell JA, Walley KR, Singer J et al (2008) Vasopressin versus norepinephrine infusion in patients with septic shock. N Engl J Med 358(9):877–887. 10.1056/NEJMoa067373 18305265

[13] Leone M, Khanna AK, Wieruszewski PM (2025) Mastering the practical application of vasopressin at the bedside in septic shock. Intensive Care Med. 10.1007/s00134-025-08204-5 41222655

[14] Uemura K, Nishikawa T, Matsushita H, et al (2024) Minimally invasive monitor of cardiac output based on the machine-learning analysis of the pulse contour of the peripheral arterial pressure. Annual International Conference of the IEEE Engineering in Medicine and Biology Society IEEE Engineering in Medicine and Biology Society Annual International Conference, pp 1–4. 10.1109/EMBC53108.2024.10781568. 40040149

[15] Matsushita H, Nishikawa T, Uemura K et al (2026) Automated hemodynamic management system for the prevention of hypotension and cardiac output reduction during general anesthesia: preclinical experimental validation. J Anesth. 10.1007/s00540-026-03720-0 41910643

[16] Uemura K, Nishikawa T, Kawada T et al (2022) A novel method of trans-esophageal Doppler cardiac output monitoring utilizing peripheral arterial pulse contour with/without machine learning approach. J Clin Monit Comput 36(2):437–449. 10.1007/s10877-021-00671-7 33598822

[17] Ganter MT, Alhashemi JA, Al-Shabasy AM et al (2016) Continuous cardiac output measurement by un-calibrated pulse wave analysis and pulmonary artery catheter in patients with septic shock. J Clin Monit Comput 30(1):13–22. 10.1007/s10877-015-9672-0 25721853

[18] Sasaki K, Kawada T, Matsushita H et al (2024) Computer-controlled closed-loop norepinephrine infusion system for automated control of mean arterial pressure in dogs under isoflurane-induced hypotension: a feasibility study. Front Vet Sci 11:1374356. 10.3389/fvets.2024.1374356 38881786 PMC11177754

[19] Bland JM, Altman DG (2007) Agreement between methods of measurement with multiple observations per individual. J Biopharm Stat 17(4):571–582. 10.1080/10543400701329422 17613642

[20] Critchley LA, Peng ZY, Fok BS, Lee A, Phillips RA (2005) Testing the reliability of a new ultrasonic cardiac output monitor, the USCOM, by using aortic flowprobes in anesthetized dogs. Anesth Analg 100(3):748–753. 10.1213/01.ANE.0000144774.42408.05 15728064

[21] Saugel B, Hapfelmeier A, Flick M et al (2025) Statistical Analysis and Reporting of Cardiac Output Method Comparison Studies (COMPARE) Statement. Anesthesiology 143(3):518–532. 10.1097/ALN.0000000000005559 40793805 PMC12625360

[22] Saugel B, Heeschen J, Hapfelmeier A, Romagnoli S, Greiwe G (2020) Cardiac output estimation using multi-beat analysis of the radial arterial blood pressure waveform: a method comparison study in patients having off-pump coronary artery bypass surgery using intermittent pulmonary artery thermodilution as the reference method. J Clin Monit Comput 34(4):649–654. 10.1007/s10877-019-00375-z 31456072 PMC7367911

[23] Saugel B, Huber W, Nierhaus A, Kluge S, Reuter DA, Wagner JY (2016) Advanced hemodynamic management in patients with septic shock. BioMed Res Int 2016:8268569. 10.1155/2016/8268569 27703980 PMC5039281

[24] Poli-de-Figueiredo LF, Garrido AG, Nakagawa N, Sannomiya P (2008) Experimental models of sepsis and their clinical relevance. Shock 30(Suppl 1):53–59. 10.1097/SHK.0b013e318181a343 18704008

[25] Permpikul C, Tongyoo S, Viarasilpa T et al (2019) Early Use of Norepinephrine in Septic Shock Resuscitation (CENSER) a Randomized trial. Am J Respir Crit Care Med 199(9):1097–1105. 10.1164/rccm.201806-1034OC 30704260

[26] Uemura K, Kawada T, Kamiya A et al (2005) Prediction of circulatory equilibrium in response to changes in stressed blood volume. Am J Physiol Heart Circ Physiol 289(1):H301–H307. 10.1152/ajpheart.01237.2004 15708956

[27] Pinsky MR (1992) Cardiovascular response in canine endotoxic shock: effect of ibuprofen pretreatment. Circ Shock 37(4):323–332 1446391

[28] Uemura K, Kawada T, Zheng C, Sugimachi M (2016) Less invasive and inotrope-reduction approach to automated closed-loop control of hemodynamics in decompensated heart failure. IEEE Trans Biomed Eng 63(8):1699–1708. 10.1109/TBME.2015.2499782 26571509

[29] Osuchowski MF, Ayala A, Bahrami S et al (2018) Minimum quality threshold in pre-clinical sepsis studies (MQTiPSS): an international expert consensus initiative for improvement of animal modeling in sepsis. Shock 50(4):377–380. 10.1097/SHK.0000000000001212 30106875 PMC6133201

[30] Persichini R, Silva S, Teboul JL et al (2012) Effects of norepinephrine on mean systemic pressure and venous return in human septic shock. Crit Care Med 40(12):3146–3153. 10.1097/CCM.0b013e318260c6c3 22926333

[31] Minneci PC, Deans KJ, Banks SM et al (2004) Differing effects of epinephrine, norepinephrine, and vasopressin on survival in a canine model of septic shock. Am J Physiol Heart Circ Physiol 287(6):H2545–H2554. 10.1152/ajpheart.00450.2004 15319205

[32] Jiang L, Sheng Y, Feng X, Wu J (2019) The effects and safety of vasopressin receptor agonists in patients with septic shock: a meta-analysis and trial sequential analysis. Crit Care 23(1):91. 10.1186/s13054-019-2362-4 30871607 PMC6419432

[33] Lamarche-Fontaneto R, Oud L, Howell KD et al (2025) Cardiac output monitors in septic shock: do they deliver what matters? a systematic review and meta-analysis. Crit Care 29(1):299. 10.1186/s13054-025-05547-9 40652247 PMC12255103

[34] Matsushita H, Kurono K, Nakabayashi M et al (2025) Non-invasive monitoring of microcirculation dynamics in hypovolemic shock: a novel application of diffuse correlation spectroscopy. Intensive Care Med Exp 13(1):53. 10.1186/s40635-025-00761-9 40450616 PMC12127257

[35] Kashihara K, Kawada T, Uemura K et al (2004) Adaptive predictive control of arterial blood pressure based on a neural network during acute hypotension. Ann Biomed Eng 32(10):1365–1383. 10.1114/b:abme.0000042225.19806.34 15535055

