# Supplementary material for: Automated hemodynamic resuscitation system for computer-controlled norepinephrine, vasopressin, and fluid therapy in endotoxin-induced shock: a feasibility and physiological proof-of-concept study
Source: Intensive Care Med Exp. 2026 Sep 20;14:124. doi: 10.1186/s40635-026-00977-3 (PMC13590466; doi:10.1186/s40635-026-00977-3)
Supplement: Supplementary file 1 [file 40635_2026_977_MOESM1_ESM.docx]

**Supplemental Material 1**

**Manuscript Title**:

Automated hemodynamic resuscitation system for computer-controlled norepinephrine, vasopressin, and fluid therapy in endotoxin-induced shock: a feasibility and physiological proof-of-concept study

**Journal:**

Intensive Care Medicine Experimental

**Authors:**

Kazumasu Sasaki, Kazunori Uemura, Hiroki Matsushita, Yuta Nakamura,

Kenta Ohba, Yasuyuki Kataoka, Takuya Nishikawa, Kei Sato, Hidetaka Morita, Masahiro Otake, Nana Hiraki, Masafumi Fukumitsu, Toru Kawada,

Kenji Sunagawa, Joe Alexander Jr, Keita Saku

**Corresponding authors:**

Kazumasu Sasaki

Akita Cerebrospinal and Cardiovascular Center,

[sasaki-kazumasu@akita-noken.jp](mailto:sasaki-kazumasu@akita-noken.jp) or k-sasaki@gaea.ocn.ne.jp

Kazunori Uemura,

National Cerebral and Cardiovascular Center,

Email: kuemura@ncvc.go.jp

**S-1-1. Circulatory Equilibrium Framework**

The system was designed based on the circulatory equilibrium framework [1, 2] as shown in the 2D diagram of cardiac output (CO, mL/kg/min) and right atrial pressure (RAP, mmHg) (Figure S1_1). In this framework, the venous return (VR) properties of the systemic vein and the apparent pumping ability of the cardiothoracic compartment are characterized by the VR curve (blue line in Figure S1) and the CO curve (red line in Figure S1_1), respectively. The CO and RAP at equilibrium can be predicted from the intersection between the CO and VR curves.

Figure S1_1. The circulatory equilibrium framework analyzed in a 2D diagram of cardiac output and right atrial pressure

Cardiac output (CO) and right atrial pressure (RAP) at equilibrium are predicted from the intersection between the CO curve (red line) and the venous return (VR) curve (blue line).

The CO curve is expressed by the following logarithmic equation:

$\text{CO = S×}\left[ \ln\left( \text{RAP-1.0} \right)\text{+0.88} \right]$　　　　 　　　　　　　　　　　(Sup 1_1)

where S is the slope of the CO curve [3, 4].

The VR curve is expressed by the following equation [2]:

$\text{VR = 7.1×}\text{V}_{\text{S}}\text{-17.9×RAP}$ 　　　　　　　　 (Sup 1_2)

where V_S_ is the systemic stressed blood volume.

At the equilibrium state, CO = VR. Hence, simultaneous solution of equations (Sup1_1) and (Sup1_2) gives the CO and RAP at equilibrium, i.e., the intersection point between the CO and VR curves (Figure S1_1). If the systemic vascular resistance (R_S_) is known, arterial pressure (AP) is predicted by the following equation:

$\text{AP = CO×}\text{R}_{\text{S}}\text{+RAP}$ 　　　　　　　　 (Sup1_3)

**S-1-2. Calculation of cardiovascular parameters in the system**

Based on the circulatory equilibrium framework, the system calculates R_S_, V_S_, and S (blue rectangle in Figure 1 in the main manuscript).

R_S_ is calculated from AP, CO (CO_es_) estimated by the minimally invasive CO monitor, and central venous pressure (CVP) as a proxy of RAP by rewriting equation (Sup1_3) as follows:

$\text{R}_{\text{S}}\text{ = }\left( \text{AP-CVP} \right)\text{/C}\text{O}_{\text{es}}$ 　　　　　　　　　 (Sup1_4)

V_S_ is calculated from CO_es_ and CVP by rewriting equation (Sup1_2) as follows:

$\text{V}_{\text{S}}\text{ = }\left( \text{C}\text{O}_{\text{es}}\text{+17.9×CVP} \right)\text{/7.1}$ 　　　　　　　　 (Sup1_5)

S can be calculated by rewriting equation (Sup1_1). However, if the CVP is reduced to the asymptotic pressure of the logarithmic function of equation (Sup1_1) or to ≤1 mmHg as observed in severe bleeding and septic vasodilation, equation (Sup1_1) no longer holds. Therefore, equation (Sup1_1) was modified in the system control as follows:

$\text{CO = S×}\left[ \ln\left( \text{CV}\text{P-1.0} \right)\text{+0.88} \right]$　 CVP >2 　　 　　　(Sup1_6a)

$\text{CO = S×}\left[ \ln\left( \text{1} \right)\text{+0.88} \right]\text{ = 0.88 }\text{× S}$　 CVP ≤2	(Sup1_6b)

Hence, S is calculated as follows,

$\text{S = CO / }\left[ \ln\left( \text{CVP-1.0} \right)\text{+0.88} \right]$　 CVP >2 　　 　　　(Sup1_7a)

$\text{S = CO / 0.88}$　 CVP ≤2	(Sup1_7b)

**S-1-3. Calculation of target cardiovascular parameters**

Based on the target AP (AP*), target CO (CO*), CO_es_, CVP, and S, the system calculates the target R_S_ (R_S_*) and target V_S_ (V_S_*) (red rectangle in Figure 1 in the main manuscript).

First, the target CVP (CVP*) is calculated as follows:

$\text{CVP* =1.0 + Exp}\left( \frac{\text{CO*}}{\text{S}}\text{-0.88} \right)$　 CVP >2 　　　　(Sup1_8a)

$\text{CVP* =CVP - 1.0 + Exp}\left( \frac{\text{CO*}}{\text{S}}\text{-0.88} \right)$	CVP ≤2	(Sup1_8b)

With CO* and CVP*, V_S_* is calculated as follows:

$\text{V}_{\text{S}}\text{* = }\left( \text{CO*+17.9×CVP*} \right)\text{/7.1}$	(Sup1_9)

R_S_* is calculated as follows:

$\text{R}_{\text{S}}\text{* = }\left( \text{AP*-CVP} \right)\text{/C}\text{O}_{\text{es}}$	(Sup1_10)

**S-1-4. The proportional-integral feedback controller of norepinephrine infusion in the system**

To minimize the difference between R_S_* and R_S_ (ΔR_S_ = R_S_*-R_S_), a proportional-integral (PI) feedback controller adjusts the infusion rate of norepinephrine (NE) (green rectangle in Figure 1 in the main manuscript). In the PI feedback controller (Figure S1_2), ΔR_S_ and the difference integrated with an integral gain (K_i_) are summed and scaled by a proportional gain (K_p_) to determine the NE infusion rate. We determined the gain constants for NE infusion (K_i_ = 0.91 min^-1^, K_p_ = 2.05 μg·kg^-1^·min^-1^ [mmHg·min·kg·mL^-1^]^-1^) based on the open-loop response of R_S_ to NE infusion determined in our previous study [5].

Figure S1_2. Block diagram of the proportional-integral controller of norepinephrine (NE) infusion. ΔR_S_ indicates the difference between R_S_ and R_S_*; Ki , integral gain constant; Kp , proportional gain constant. s is a Laplace operator.

In the study [5], we examined the R_S_ response to the infusion of NE in three dogs with endotoxin-induced shock. Surgical preparation including placement of the aortic flow probe, and measurements of AP, reference CO (CO_ref_) and CVP were performed as described in the text. Under open-loop condition, we infused NE at 0.3 μg·kg^-1^·min^-1^ for 10 min to obtain a step response of R_S_ [5]. We described the step response by a transfer function of a first-order model with a transport delay. In this model, the change in R_S_ from baseline (δR_S_) in response to NE infusion can be expressed by the following formula:

$\text{δ}\text{R}_{\text{S}}\text{(t) =}\left\{ \begin{matrix} \text{G}\text{⋅}\left[ \text{1-}\exp\left( \text{-}\frac{\text{t-L}}{\text{T}} \right) \right]\text{ }\left( \text{t ≥ L} \right) \\ \text{0 }\left( \text{t < L} \right) \end{matrix} \right.$	(Sup2_1)

where G is static gain [mmHg·min·kg·ml^-1^ (μg·kg^-1^·min^-1^)^-1^], L is transport delay (s), and T is time constant (s). We estimated the parameters G, L, and T by approximating δR_S_ to Eq. sup2_1 using the least square method [5]. The averaged values of G, L, and T were 0.33 mmHg·min·kg·ml^-1^ (μg·kg^-1^·min^-1^)^-1^, 79 s, and 56 s, respectively. Using these parameters and empirical tuning, we defined the gain constants, Ki = 0.91 min^-1^ and Kp = 2.05 μg·kg^-1^·min^-1^ (mmHg·min·kg·ml^-1^)^-1^, for the proportional-integral (PI) feedback controller of NE infusion (Figure S1_2).

**S-1-5. Controllers of infusion of vasopressin and fluid in the system**

Based on AP and the NE infusion rate, a non-linear feedback controller (magenta rectangle in Figure 1 in the main manuscript) adjusts infusion rate of vasopressin (AVP) every 5 min (at 0, 5, 10 min, etc.) based on the following “if-then” rules:

If the infusion rate of NE is >1.0 μg·kg^-1^·min^-1^, then infuse AVP at 0.04 IU·min^-1^.

If NE and AVP are concomitantly infused, AP > AP* - 5 mmHg, and the infusion rate of NE <0.5 μl·min·kg^-1^, then reduce the infusion rate of AVP in a stepwise manner (0.04 → 0.02 → 0.013 → 0.01 IU·min^-1^). If the AVP infusion rate has already been reduced to 0.01 IU·min^-1^, then quit the infusion of AVP. NE threshold for initiating AVP (1.0 μg·kg^-1^·min^-1^) is higher than the threshold suggested by the SSC guidelines (0.25-0.5 μg·kg^-1^·min^-1^) [6]. This higher threshold is chosen because our previous study using canine endotoxin shock models [5] demonstrated that substantially higher doses of NE were required to achieve adequate hemodynamic resuscitation.

To minimize the difference between V_S_* and V_S_ (ΔV_S_ = V_S_*-V_S_), an on/off feedback controller (orange rectangle in Figure 1 in the main manuscript) adjusts the infusion rate of Ringer’s acetate solution (RiA) based on another set of “if-then” rules. Specifically, if V_S_ ≤ V_S_*, then RiA is infused at a rate of 800 mL·h^-1^. If V_S_ > V_S_*, then the infusion is stopped.

**References for Supplemental Material 1**

1. Guyton AC (1955) Determination of cardiac output by equating venous return curves with cardiac response curves. Physiol Rev 35:123–129. <https://doi.org/10.1152/physrev.1955.35.1.123>

2. Uemura K, Sugimachi M, Kawada T et al (2004) A novel framework of circulatory equilibrium. Am J Physiol Heart Circ Physiol 286:H2376–2385. <https://doi.org/10.1152/ajpheart.00654.2003>

3. Uemura K, Kamiya A, Hidaka I et al (2006) Automated drug delivery system to control systemic arterial pressure, cardiac output, and left heart filling pressure in acute decompensated heart failure. J Appl Physiol 100:1278–1286. <https://doi.org/10.1152/japplphysiol.01206.2005>

4. Uemura K, Kawada T, Kamiya A et al (2005) Prediction of circulatory equilibrium in response to changes in stressed blood volume. Am J Physiol Heart Circ Physiol 289:H301–307. <https://doi.org/10.1152/ajpheart.01237.2004>

5. Uemura K, Kawada T, Zheng C, Li M, Sugimachi M (2017) Computer-controlled closed-loop drug infusion system for automated hemodynamic resuscitation in endotoxin-induced shock. BMC Anesthesiol 17:145. <https://doi.org/10.1186/s12871-017-0437-9>

6. Prescott HC, Antonelli M, Alhazzani W, et al (2026) Surviving sepsis campaign: international guidelines for management of sepsis and septic shock 2026. Crit Care Med 54(4):725–812. https://doi.org/10.1097/CCM.0000000000007075
