# Supplementary material for: Automated hemodynamic resuscitation system for computer-controlled norepinephrine, vasopressin, and fluid therapy in endotoxin-induced shock: a feasibility and physiological proof-of-concept study
Source: Intensive Care Med Exp. 2026 Sep 20;14:124. doi: 10.1186/s40635-026-00977-3 (PMC13590466; doi:10.1186/s40635-026-00977-3)
Supplement: Supplementary file 2 [file 40635_2026_977_MOESM2_ESM.docx]

**Supplemental Material 2**

**Manuscript Title**:

Automated hemodynamic resuscitation system for computer-controlled norepinephrine, vasopressin, and fluid therapy in endotoxin-induced shock: a feasibility and physiological proof-of-concept study

**Journal:**

Intensive Care Medicine Experimental

**Authors**:

Kazumasu Sasaki, Kazunori Uemura, Hiroki Matsushita, Yuta Nakamura,

Kenta Ohba, Yasuyuki Kataoka, Takuya Nishikawa, Kei Sato, Hidetaka Morita, Masahiro Otake, Nana Hiraki, Masafumi Fukumitsu, Toru Kawada,

Kenji Sunagawa, Joe Alexander Jr, Keita Saku

**Corresponding author**:

Kazumasu Sasaki

Akita Cerebrospinal and Cardiovascular Center,

[sasaki-kazumasu@akita-noken.jp](mailto:sasaki-kazumasu@akita-noken.jp) or k-sasaki@gaea.ocn.ne.jp

Kazunori Uemura,

National Cerebral and Cardiovascular Center,

Email: kuemura@ncvc.go.jp

**S-2. Experimental time courses in individual animals**

Experimental time courses of automated hemodynamic resuscitation in each of the 6 dogs over a 60 min period. (**A**) Time courses of norepinephrine (NE) and vasopressin (AVP) infusion and cumulated volume of Ringer’s acetate solution (RiA) infused. (**B**) Time courses of cardiovascular parameters. Red lines indicate target parameters (top; target vascular resistance [RS]*, bottom; target stressed blood volume [VS]*).). (**C**) Time courses of hemodynamic variables. Red lines indicate target hemodynamic variables (top; target arterial pressure [AP*], second from the top; target cardiac output [CO*]). (**D**) Time course of the reference CO (CO_ref_). The AP* and CO* were constant throughout the control period, while the R* and V* were recalculated every minute.

**Animal #1**

**Animal #2**

**Animal #3**

**Animal #4**

**Animal #5**

**Animal #6**
